# Can we now save the neck of OSCC T1–T2 patients? A narrative review of whether experimental techniques are on the way to clinical application

**DOI:** 10.37349/etat.2026.1002384

**Published:** 2026-07-23

**Authors:** Vincenzo Cuccurullo, Graziella Di Grezia, Gianluca Gatta, Giuseppe Lucio Cascini

**Affiliations:** IRCCS Istituto Romagnolo per lo Studio dei Tumori (IRST) “Dino Amadori”, Italy; ^1^Department of Precision Medicine, University of Campania “Luigi Vanvitelli”, 80138 Napoli, Italy; ^2^Department of Life Sciences, Link Campus University, 00165 Roma, Italy; ^3^Department of Medicine and Health Science, University of Molise, 86100 Campobasso, Italy

**Keywords:** squamous cell carcinoma, cervical lymph node staging, sentinel node, fluorescence, radiomics, narrative review

## Abstract

The molecular pathogenesis of oral squamous cell carcinoma (OSCC) is a complex process involving genetic alterations that accumulate over time. The clinical presentation and management of patients vary according to several pathological factors, but lymph node involvement is a key consideration in this context. Accurate lymph node staging in the neck is challenging because metastases may be present at an early stage of oral cancer. This narrative review assesses the evolution of imaging techniques over the last five years, distinguishing between research and established methods in order to address specific clinical questions. Conventional imaging techniques, including ultrasonography, contrast-enhanced computed tomography, contrast-enhanced magnetic resonance imaging and fluorine-18 fluorodeoxyglucose positron emission tomography, are reviewed and compared with several noteworthy developments in optical and fluorescence imaging. In this scenario, various position papers and guidelines strongly suggest using scintigraphy for sentinel node biopsy. However, its use in clinical practice is not widespread. This is a direct result of unrelenting advances in imaging accuracy, treatment options and new diagnostic approaches. All these approaches are considered here, but none emerges as a universal solution. Overall, no single technique currently emerges as a universal solution. The most realistic strategy is an integrated, multidisciplinary pathway. Multimodal imaging integration framework and AI-assisted diagnostic strategy could provide a novel clinical decision-making pathway for cervical lymph node staging in T1–T2 stage OSCC patients.

## Introduction

Over the past five years, there has been a significant increase in the understanding of the mechanisms of tumour spread in patients with oral squamous cell carcinoma (OSCC). Many pathological findings have highlighted the role that primary tumour behaviour plays as a prognostic factor. At the same time, the development of new applications and imaging techniques has increased the number of tools available for correctly stage these patients. However, the preoperative detection of neck nodal metastases remains challenging, especially in T1/T2 lesions. This narrative review aims to evaluate how established and new techniques can be combined in light of the ever-changing clinical scenario of OSCC.

## The changing scenario of OSCC

OSCC is a relatively common malignancy worldwide, accounting for the majority of oral cancers, with higher incidence in older males and strong associations with tobacco and alcohol use [1]. OSCC originates from the lining epithelium of the oral cavity and is characterized by its ability to develop in any area of the mouth, including the lips, tongue, gums, palate, and cheeks. It accounts for around 90% of all malignant neoplasms that develop in the oral cavity [2]. The epidemiology of OSCC shows clear geopolitical differences: incidence is highest in South and Southeast Asia due to widespread use of betel quid, tobacco, and alcohol, while in Western countries rates are lower but increasing; additionally, limited healthcare access in low- and middle-income regions leads to later diagnosis and worse outcomes compared to high-income countries [3]. Furthermore, it is hypothesised that genetic mutations may be a causative agent in carcinogenesis [4], driven by the accumulation of genetic alterations affecting key tumor suppressor pathways, among which *TP53* and *NOTCH1* are central. Mutations in *TP53*—frequently observed in OSCC—lead to loss of these protective functions, allowing the survival and proliferation of genetically damaged cells. Genomic studies have shown that *NOTCH1* mutations occur in a substantial proportion of OSCC cases and may arise early in premalignant lesions, supporting a driver role in tumor initiation and progression. Additionally, both *TP53* and *NOTCH1* are frequently co-altered within broader oncogenic pathways in OSCC, highlighting their importance as biomarkers and potential therapeutic targets in this disease [5]. Although the potential for OSCC to develop without premalignant tissue changes remains unclear, it has been shown that at least 20% of OSCCs originate from clinically visible precursor lesions [6].

OSCC manifests itself in a heterogeneous way, including as ulcers, exophytic vegetations, or as a combination of these. The neoplastic epithelium is squamous in well-differentiated types and is characterised by islands of malignant keratinocytes and intercellular bridges and corneal pearls [7]. Mitoses are frequently observed, with a significant proportion of these being atypical. These histological variations mirror tumour heterogeneity, which can manifest in diverse microscopic forms [8]. These features can influence prognostic outcomes and therapeutic strategies [9].

The management of early-stage (T1–2 N0) OSCC involves the surgical excision of the primary tumour. There are two options: the upfront elective neck dissection (END) or the watchful waiting involving clinical and radiological surveillance, followed by therapeutic neck dissection in the event of neck nodal recurrence [10]. A substantial body of evidence from a range of direct meta-analyses of randomised clinical trials supports the superiority of END over alternative approaches. This is particularly evident in patients diagnosed with early-stage OSCC (N0), with regard to nodal recurrence, overall survival and disease-specific survival [11].

The status of the neck significantly influences the clinical outcome; the presence of metastases in the laterocervical lymph nodes is, in fact, the most important prognostic factor. The five-year survival rate ranges from 82% to 53%, depending on the node stage [12].

Neoadjuvant chemotherapy (NACT) has been proposed as a way of avoiding these negative effects. However, NACT has primarily been researched as a treatment for head and neck cancers to reduce surgical margins and rates of distant metastasis, thereby improving outcomes [13].

It is worth noting that lymph node involvement can occur at an early stage of oral cancer, but its detection can be difficult to assess [14]. Regional lymph node enlargement may be due to specific hyperplastic responses and require further evaluation. Conversely, a node with normal dimensions may be within a tumour invasion [15]. All of this evidence has implications for patient management and surgical choice; indeed, lateral cervical drainage may not always be the optimal strategy [16]. The aforementioned surgical procedure is associated with a high incidence of postoperative complications, which can have a significant impact on both aesthetic outcomes and functional capacity [17].

Mapping lymphatics is an alternative approach to managing OSCC patients. Sentinel lymph node biopsy (SLNB) has been shown to significantly reduce mortality rates, operative time and hospitalisation duration compared with latero-cervical dissection [18].

A notable benefit of this method is its ability to provide a more precise staging of lymphatic drainage in patients with clinically negative (N0) neck nodes, thereby restricting latero-cervical emptying.

The low rate of detecting microscopic metastatic foci during standard histological assessment increases in selected SLN basins. This is one of the most compelling arguments for SLNB [19].

In recent years, technological advances in lymphatic system imaging have been incessant because the choice of imaging modality and surgical technique has an impact on disease-free survival and overall survival [20].

In this scenario, it is far from possible to obtain univocal approaches; this may be attributed to variable imaging accuracy, emerging new diagnostic procedures and continuously changing treatment options. Only by addressing these issues together will we be aware of the possible choices and clinical opportunities.

Moreover, social and economic issues affect diagnostic algorithms: from a public-health perspective, patients with OSCC living in rural or medically underserved areas could plausibly benefit from national programmes that strengthen referral pathways, imaging access, multidisciplinary review, and follow-up capacity. Recent data in head and neck cancer show that rural residence is associated with longer time to treatment and higher acute-care utilization, while the World Health Assembly has recently urged Member States to expand equitable medical imaging capacity through coordinated policies, infrastructure investment, and workforce training. In this context, decentralizing selected diagnostic steps while preserving referral to expert centres for complex decisions may improve access, reduce delays, and lessen pressure on high-volume tertiary institutions.

On scintigraphy, the key issue is organizational as much as diagnostic. Radiotracer-guided SLNB in oral cancer requires close coordination among nuclear medicine, radiology, surgery, and pathology, and EANM guidance states that Single Photon Emission Computed Tomography combined with Computed Tomography scans (SPECT/CT) is mandatory when available because it improves anatomic localization of sentinel nodes. This makes the pathway dependent on radiopharmacy access, scanner availability, trained personnel, and tight perioperative scheduling, all of which are harder to guarantee outside high-volume centres. In addition, SLNB has a recognized learning curve, and accuracy is lower in floor-of-mouth tumours because the injection site can mask adjacent nodes through the “shine-through” effect.

## Where does the standard imaging stand?

In recent years, we have observed a paradox in the evaluation of the neck in patients with oral cancer: there is more interest in pathology to predict the nodal status from the primary than in the imaging contribution to its direct evaluation. This may be explained by low clinician confidence in the negative predictive value (NPV) of imaging techniques.

Standard imaging methods are ultrasonography (US) [21], contrast-enhanced computed tomography (CECT) [22], contrast-enhanced magnetic resonance imaging (CE-MRI) [23], and fluorodeoxyglucose positron emission tomography (FDG-PET) [24]. Image-guided biopsy or fine-needle aspiration cytology is also essential for tissue diagnosis, especially in deeper lesions [21, 22].

CECT has a primary role in the diagnosis and staging of OSCC, except when the tumour is located in the tongue or floor of the mouth. In these cases, CE-MRI is the imaging modality of choice. CECT plays a pivotal role in the local staging of gengivo-buccal OSCC because it belongs high accuracy for detection of depth of invasion, bone erosion and peri-neural involvement, if performed with puffed cheek technique [22].

CECT and FDG-PET accurately detect the sites of distant metastases in lungs, liver, bones, and mediastinal nodes. Conversely, cervical lymph node staging is more challenging, as US, CT, PET and MRI have shown a wide range of accuracy depending on tumour heterogeneity; nodal involvement is present in almost 50% of floor of mouth and retromolar trigone lesions, whereas it is rare in oral mucosal or hard palate disease [25]. The potential for OSCC to spread to the lymphatic system has implications for managing patients, as this is thought to reduce survival by 50%.

Lymphatic drainage patterns are typically sequential from level I, but skip metastases have been observed in anterior tongue lesions. The high rate of occult lymph node metastases (LNMs) has justified prophylactic or END. END from level I to III is recommended in the clinically N0 neck as it improves overall and disease-free survival compared to initial surveillance [26].

In a more aggressive approach (radical neck dissection), the spinal accessory nerve, sternocleidomastoid muscle and internal jugular vein are sacrificed. It is mandatory when extracapsular or extra-nodal extension (ENE) is observed. However, these considerations are based on the ability of clinical imaging to accurately stage patients with nodal disease and select those who are truly negative [27].

The choice of imaging technique for neck evaluation is based on two cornerstones: the imaging technique used to stage the primary site should be the same as that used to stage nodal involvement; in cases of doubt, US with or without FNAC or FNAB is strongly indicated. According to this last sentence, the US plays an important role in the management of the neck of OSCC patients, although it is more practical than evidence-based. US helps clinicians identify an enlarged lymph node and the likelihood that it is malignant. However, clinical N0 OSCC is assumed if clinical examination, CT or MRI don’t show any enlarged or abnormal nodes; US-FNA is used in equivocal findings and must be negative in this group of patients. Clearly, defining the criteria for malignancy is critical to the proper management of these patients.

These are generally standardised, but vary in the order of imaging techniques used and the location or level of the lump in the neck [28].

For example, in a 2025 article, the criterion for lymph node positivity on US images was a short diameter ≥ 8 mm in levels I and II, ≥ 6 mm in other regions; the same diameter must be ≥ 10 mm on CT and MRI images. Additional criteria were morphology (presence of a hilum and long/short ratio) and internal condition (heterogeneity or internal necrosis) [29].

When US is used, other criteria include the presence of abnormalities or defects on Doppler analysis with blood flow signals at the margins. The stiffness compared to the surrounding tissue on strain elasto-sonography may be useful.

The size, shape, echogenicity, echogenic hilum, internal echo, necrosis, margin, vascular pattern and elastography represent the main features associated with malignancy in US imaging.

Lymph node size is clearly one of the more useful features, but different cut-offs have also been proposed according to neck level. Shape is easier to standardise: the ratio between short and long-axis diameters (S/L ratio) describes the tendency to malignancy well, since round nodes with an S/L ratio > 0.5 are more likely to be malignant [30].

Other malignant features include hypoechoicity with disappearance of the echogenic hilum, heterogeneity of the internal echo, sometimes necrosis and irregular margins; groups of nodes may be seen in extensive lymphatic spread. Lymph node elasticity may be assessed by elasto-sonography as it may be reduced in affected nodes.

All these parameters are often combined in a unique scoring system to predict cervical node involvement; the various proposed models have achieved good sensitivity (90%) and specificity (85%), but in clinical practice they are influenced by the skill of the operator, not necessarily a specialist in diagnostic imaging.

MRI is the imaging modality of choice for local assessment of OSCC because of its very high soft tissue contrast. This allows the extension of the primary cancer to be well staged. There is little data on its impact on the detection of nodes in the neck [31]. The MRI parameters used to characterise nodules are the same as those used in conventional imaging: shape, size, extracapsular extension and abnormal internal architecture. Size and shape are certainly valid criteria for the diagnosis of massive metastatic invasion, but they may fail in the case of partial invasion or micro-metastasis. The presence of central necrosis and conglomeration is typical of massive lymphatic spread [32]. Non-morphological MRI sequences such as diffusion weighted imaging (DWI) can be helpful in detecting nodal metastases. Baseline and post-contrast MRI, in fact, allows to characterise the shape and dimensions of a nodule, as well as the cellularity and diffusion of water and vascularisation, in the same plane (transaxial and coronal both), in the same voxels. Much attention has been paid to the role of ADCs as a single value related to the number of cells in a lesion. In general, restricted diffusion is associated with high cell counts and lower ADCs. A cut-off value of 1.03 × 10^–3^ mm^2^/s is usually considered to be able to discriminate between benign and malignant nodules. However, this is not widely accepted due to lack of standardisation [33].

CECT, along with MRI, is considered the standard diagnostic modality for staging the primary and neck of patients with OSCC. Many guidelines support this, but the proposed criteria for detecting involved lymph nodes are variable, as different radiological features may indicate a suspected LN. CT of non-involved nodes typically shows soft tissue density, kidney-shaped morphology with fat in the hilum. Metastatic, on the other hand, appear round with irregular margins and central hyperdensity; peripheral contrast enhancement is usually present and uneven in the central part. The size remains a commonly used criterion, but it ranges between 5 and 15 mm.

For example, in a 2023 study of CECT for nodal detection in OSCC, lymph nodes were categorised according to short-axis diameter as accelerated (< 10 mm), enlarged (≥ 10 mm), and melted. The first group was considered when the nodes appeared slightly enlarged in side-by-side comparison, but remained less than 1 cm in size. “Melted” was identified in the presence of necrosis as a hypodense central area surrounded by an enhancing irregular rim of tissue. Notably, the authors observed higher detection of LNMs in level IIa and IIb, where 88.46% and 92.86% were accentuated, 76.92% and 78.57% were enlarged, and 53.85% and 57.14% were melted. Conversely, in level III, only 62.96% of LNMs were enlarged on CT, and merely 44.44% were melted. Few cases were in level I. The accentuated nodes had a sensitivity of 83.54% but a low specificity (55%) and positive predictive value (PPV) (47%); The group of enlarged nodes showed lower sensitivity (65%) but higher specificity (84%) and PPV (67%). The NPV reached similar values for both groups (around 85%). In this large series of 239 OSCC patients, the authors reported 13% of false-negative CT scans, although they also classified the group of accelerated nodules as suspicious in the analysis. It is important to note that including the group of accelerated nodes, the risk of overestimating neck staging is approximately 50% [34].

However, few studies have compared the diagnostic performance of US, CECT, MRI and PET in the same series of patients: a 2024 study did this and found lymph node-based accuracies of 96.9%, 96.7%, 96.2% and 93.6%, respectively. In this series of 16 patients with tongue and oral cavity cancer, metastases were detected in only 22 of 424 nodes examined, with PPV and NPV of 81.8% and 87.3% for US, 76.9% and 88.4% for CT, 72.7% and 85.9% for MRI, respectively. 51.9% and 92.7% for PET [35]. This experience has the advantage of providing a direct comparison between imaging methods on a lymph node basis, because it is very difficult to localise during surgery, the corresponding node visualized on imaging. In addition, it is difficult to identify the exact plane of a lymph node detected by the US on CT or MRI. However, the imaging accuracy and performance are comparable, although the study was limited by the small number of patients, the ratio of metastatic nodes, and the high rate of mismatched lesions. In addition, a large number of nodes were stage I.

The issue of lymph node staging in the neck of OSCC, as mentioned above, has mainly been addressed by using high resolution techniques; although FDG is claimed to be an optimal agent for tumour detection, PET has been hampered by low spatial resolution. FDG-PET has been widely used for staging head and neck tumours and has a high sensitivity for lymph node detection, as shown in some reports published at the end of the last century [36–38]. However, the accuracy of PET is related to nodal size, as shown by Yamazaki and colleagues: they demonstrated 100% tumour detection for lymph nodes > 1 cm in diameter, but none of the involved nodes < 5 mm were correctly detected [39]. This aspect is of great importance in a clinical scenario where more than 50% of metastatic lymph nodes are less than 1 cm in diameter. More recently, the introduction of fully digital PET scanners has partially overcome the resolution limitation, allowing better lymph node staging in this patient population. A study published in 2022 by Kojima et al. [40] compared non-digital to digital PET in metastatic lymph nodes and reported an increase in sensitivity and specificity from 68.2% and 89.3% to 86.2% and 95.6%. They achieved a significant increase in accuracy for nodes > 1 cm in diameter, between 0.5–1 cm and < 0.5 cm. In addition, the SUV max value remained similar for all three groups of nodes. Therefore, the SUV cut-off values obtained in digital PET, can be used regardless of diameter [40].

## The standard pre surgical techniques

Lymphatic mapping is considered to be the gold standard for the identification of SLNs [41]. The standard method involves the combination of blue dye and a radiopharma labeled with technetium-^99m^ [42]. Recent technological advances have led to the development of real-time imaging probes, which have become indispensable tools in the field of surgical assistance. The advent of novel methodologies was driven by a series of logistical challenges, namely the availability and management of radioisotopes, the infrastructure required for nuclear medicine facilities, costs, and the occurrence of anaphylaxis due to the use of blue dye [43].

The advent of ^99m^Tc-Tilmanocept as a radiotracer for SLNB has engendered a more precise instrument for SLNs identification in early-stage oral cancer; the underlying mechanism of this effect is its receptorial nature [44].

Tilmanocept is a CD206 receptor-targeted radioisotope tracer that is selectively retained in the reticuloendothelial cells of sentinel lymphatic node sites. Its advantageous physical properties include a low molecular weight and rapid clearance following injection [45].

In recent years, developments in technology in the field have led to the incorporation of novel tracers into clinical practice, namely indocyanine green (ICG), superparamagnetic iron oxide (SPIO), and microbubbles.

Fluorescence imaging with ICG facilitates the detection of SLNs for direct visual identification during surgery. ICG is a biocompatible near-infrared (NIR) contrast agent used in medical imaging because, when excited by external 750–800 nanometre wavelength light, it produces longer wavelength NIR light, allowing various tissues and organs to be accurately visualised. This is of particular value for surgeons that using a NIR camera can visualize in real time the subcutaneous lymphatic flow [46].

In comparison to methylene blue, ICG is not discernible to the naked eye but is fluorescent under NIR, generates minimal background signals, and is not absorbed by surrounding tissues.

Sugie et al. [47] conducted a systematic review of publications on 12 studies and 1,736 patients to evaluate the diagnostic performance of ICG fluorescence imaging compared with the standard radioisotope technique. Their results confirmed that the ICG fluorescence method is a useful alternative to the standard method for SLNB [47].

A highly-cited meta-analysis by Garau et al. [48] compared ^99m^Tc-nanocolloid planar lymphoscintigraphy with intra-operative use of hand-held gamma-probe, additional contribution of pre-operative SPECT/CT, and additional intraoperative contribution of optical tracers. The meta-analysis extrapolated 3,693 cases from 1,379 articles. The authors concluded that SPECT/CT is superior to planar scintigraphy in identifying SLNs from aberrant drainage and facilitating more effective planning for operations. SPECT/CT with blue dye is no more effective than radiocolloid-based methods [48].

SPIO, or superparamagnetic iron oxide, is a magnetic tracer injected subcutaneously around the lesion. It identifies the SLNs within minutes by way of iron deposition in the sinuses and macrophages. Subsequently, the SLNs are identified with a handheld magnetometer that is responsible for generating an alternating magnetic field, the purpose of which is to magnetize the SPIO particles in the proximity of the probe [49]. In a multicenter prospective study, Karakatsanis et al. [50] compared the efficacy of SPIO as a tracer in SLNB in breast cancer with radioisotopes and patent blue, recruiting patients in 7 hospitals. They also performed a meta-analysis of all published studies. This study’s findings indicate that there are no statistically significant differences in the rates of detection, suggesting that the presence or absence of metastases does not influence the efficacy of the method under scrutiny [50].

A hybrid tracer, ICG-^99m^Tc-nanocolloid (both radioactive and fluorescent) was developed to address relative limitations [51]. In a more recent systematic review and meta-analysis, Wang et al. [52] compared the differences between the novel hybrid tracer and conventional methods using ICG or radioisotope for SLNB in head and neck malignancies. The utilization of a hybrid tracer has been demonstrated in this report to yield a higher detection rate in SLN biopsies of head and neck malignancies when compared to the use of ICG or Radioisotopes. While there was no statistically significant difference in the detection rate of SLNs between ICG and radioisotopes, the use of either modality alone may often be limited in practice [52].

In a 2025 newly published article, Liu et al. [53] from a Chinese group presented the first study on the efficiency of ^99m^Tc‑rituximab for SLN mapping and biopsy in OSCC. Rituximab, a chimeric monoclonal antibody, has been shown to target the CD20 antigen, which is present on the surface of pre-B and mature B lymphocytes. ^99m^Tc-rituximab has been developed as an SLN tracer that utilizes the CD20 antigen, which is found in high concentrations in LNs. In this report, using Rituximab as an imaging agent, the SLN detection rate was 91.3% (21/23 patients) for SLN SPECT preoperative imaging and 100% (23/23) for intraoperative detection using a hand-held camera, with no statistically significant difference. In this study, they conducted a follow-up on the patients; during the designated follow-up period, no patients exhibited LNM (100.0%) [53].

However, due to the intricate anatomy of the head and neck region, the presence of numerous substantial anatomical structures, the region’s variable drainage patterns, and the frequent location of SLNs in close proximity to the primary tumor site, SLNB of the head and neck region is often more challenging than that of other anatomical sites.

Table 1 summarizes the main imaging techniques used for staging cervical lymph nodes in early-stage oral cancer. It compares each modality—such as US, CT, MRI, and PET/CT—based on their key features, strengths, limitations, diagnostic performance, and availability.

**Table 1:** Standard imaging modalities for cervical lymph node staging in oral squamous cell carcinoma (OSCC) (T1–T2 cN0).

**Modality**	**Key features**	**Strengths**	**Limitations**	**Diagnostic performance**	**Availability**
**Ultrasonography (US)**	Morphology, Doppler flow, elastography	No radiation; real-time; FNAC guidance	Operator-dependent; limited deep-node assessment	Sensitivity ~90% Specificity ~85%	High
**Contrast-enhanced computed tomography (CECT)**	Size, shape, enhancement	High spatial resolution; deep nodal stations; wide availability	Size-based criteria; poor micrometastasis detection	Sensitivity 65–84% Specificity 55–98%	High
**Contrast-enhanced magnetic resonance imaging (CE-MRI)**	High soft-tissue contrast; diffusion weighted imaging (DWI)/ADC	No ionizing radiation; multiparametric evaluation	Cost; acquisition time; ADC variability	Sensitivity ~79% Specificity ~88%	Medium
**Fluorodeoxyglucose positron emission tomography (FDG-PET)/CT**	Glucose metabolism (SUV)	Whole-body staging; detection of occult disease	Limited spatial resolution; inflammatory uptake	Sensitivity 85–90% Specificity 90–95%	Medium

## Innovative approaches in well established imaging techniques

The previous sections have highlighted the improvements that have been made in imaging in recent years, but there are still a number of areas where staging of OSCC is unsatisfactory. Main drawbacks in nodal staging can be attributed to 2 different areas: the high rate of involvement of nodes with normal dimensions and the heterogeneity of lymphatic spread. These two problems often coexist, but their impact is accentuated by the lack of prospective studies and very strict guidelines. The idea of diagnostic imprecision due to the inability of the human eye to detect small details, explains the call for AI in this clinical scenario. Radiomics and deep learning may be the solutions to show very small variations of a signal, but they require large numbers to automate procedures and analysis. In this context, the first radiomic approaches were applied to CT because of its wide availability and high image quality, which facilitate segmentation and feature extraction. Subsequently, MRI has been advocated to improve analysis due to its higher resolution in contrast, soft tissue representation, and number of parameters that can be extracted. To reduce analysis time and intra-observer variability, more sophisticated and automated approaches have been proposed using AI. The combination of deep learning and MRI-based radiomics represents a significant step forward in the automation process. The idea behind this process is to automatically detect MRI patterns that correlate with metastatic involvement of lymph nodes in the neck. Recently, Wang et al. [54] have demonstrated that machine learning using a regression analysis (LASSO) performs better than conventional diagnostic criteria. The radiomic analysis was performed in 120 OSCC patients after lymph node segmentation on post-contrast T1 images, also measuring trans-axial diameter and ADC index; thus they have observed a statistically significant only for 8 out of 272 extracted features in predicting lymph node involvement. Radiomic signatures predicted the involved node with an accuracy of 79%, improving on DWI-ADC’s results (55%) and node dimensions (73%).

MRI is the most studied technique for AI analysis because of its ability to superimpose different morphological and functional sequences, such as T1-weighted, T2-weighted and DWI and post-contrast phases. This provides a multi-parametric image where, for example, a lymph node corresponds to a group of voxels in which different aspects of its structure are present together but can be selectively analysed. The large amount of data requires high computational power to extract specific signatures for tumour detection, train the model and ultimately automate the process. However, some AI-based studies have been proposed for nodal prediction in head and neck cancer, but some proposed features were extracted from manually drawn ROIs, while others used deep learning algorithms to extract them; in the latter case, the data was generally sent to neural networks to perform classification. These techniques are widely used in medical research, but only 23 papers have applied radiomics and deep learning to diagnostic images for the preoperative diagnosis of LNM in head and neck tumours, according to a 2025 meta-analysis published by Parya Valizadeh and colleagues [55]. The papers were selected after a quality assessment and were based on CT (12), MRI (6), PET/CT (3) and US (2). Most of them extract radiomic features after manual segmentation; deep learning was used in only 7 of them. The review reports differences in the diagnostic accuracy according to the image-based model, which is higher for PET (92%) and CT (91%) and lower for MRI (84%). However, the CT-based papers were negatively affected by a high degree of heterogeneity, with variable results in terms of sensitivity and specificity, with some outliers. When they were excluded from the analysis, PET-CT models were the most accurate. Looking at the differences between deep learning and handcrafted methods, the authors found that the accuracies were very similar, 92% and 91% respectively, but noted that in all cases there was no external validation of the results [55].

Another recent meta-analysis focusing on SLN prediction in OSCC using AI-based techniques was published by Deng et al. [56]. They selected only 14 studies out of 219 examined, based on CT or MRI images and histopathological confirmation. In 5 studies, the AI analysis was based on MRI, while in 9 studies it was based on CT. Twelve studies used manual feature extraction. Only 7 reports included an external validation of the results. All selected studies were retrospective. The pooled accuracy of AI was 93%, which is consistent with other results, although they were performed in head and neck tumours, as the article cited above [55]. AI-based techniques have higher accuracy in predicting nodal involvement in OSCC compared to experienced radiologists (93% vs. 81%). However, it is worth noting that none of the studies are prospective and the results appear to be particularly granular. It is therefore striking that AI techniques in this context are not yet robust enough to enter daily clinical practice [56] and their clinical translation remains limited by the methodological fragility of the available evidence.

The first bottleneck is data heterogeneity. Current models are trained on datasets generated with different scanners, acquisition protocols, reconstruction settings, segmentation strategies, annotation habits, and reference standards. In addition, the biological target is not uniform across studies, because some algorithms are trained on primary tumour regions of interest, whereas others are trained on individual lymph nodes or mixed imaging-clinical features. This variability can inflate performance within a single centre while markedly reducing transportability across institutions. In the recent head and neck meta-analysis by Valizadeh et al. [55], 23 studies met the inclusion criteria and the pooled AUC was 91% for CT-based radiomics, 84% for MRI-based radiomics, and 92% for PET/CT-based models; however, the authors also stressed the substantial heterogeneity of the literature and the fact that most analyses had to be confined to internal validation sets because external validation was largely absent.

A second bottleneck is limited model generalization. Many published studies are retrospective, single-centre experiences based on relatively small or imbalanced cohorts, often relying on manual segmentation and cross-validation-heavy workflows. Under these conditions, the risk of overfitting, information leakage, and optimistic performance estimates becomes substantial. This concern is reinforced by the OSCC-focused systematic review and meta-analysis by Mohideen et al. [57], which included 40 studies, of which 33 entered quantitative synthesis. The pooled sensitivity and specificity of AI models for LNMs prediction were 0.86 and 0.91, respectively, suggesting real promise, yet the same review documented substantial heterogeneity, significant publication bias, and only two multicentre investigations. Therefore, high pooled accuracy should not be interpreted as proof of clinical readiness, especially when robustness across institutions, scanners, and patient subgroups remains uncertain.

A third bottleneck concerns interpretability, workflow integration, and regulatory approval. An algorithm that may influence nodal staging or the choice between END, surveillance, and sentinel-node strategies is not only a research tool, but a potential software medical device. For this reason, translation requires more than retrospective discrimination metrics: it requires analytical validity, clinical association and performance, predefined intended use, human oversight, and evidence that performance remains stable after deployment. International and regulatory frameworks already point in this direction [58]. The IMDRF clinical evaluation framework for Software as a Medical Device explicitly links approval to valid clinical association, analytical validation, and clinical validation [59]. In the United States, the FDA notes that AI/ML medical devices are reviewed through established premarket pathways and emphasises careful management across the total product life cycle, while recent Good Machine Learning Practice guidance highlights the need for safe, effective, and high-quality AI-enabled devices [60]. In Europe, MDCG 2025-6 clarifies the joint application of the Medical Devices Regulation (MDR)/In Vitro Diagnostic Medical Devices Regulation (IVDR) and the AI Act for high-risk medical device AI, with requirements extending to lifecycle management, data governance, risk management, human oversight, conformity assessment, and post-market monitoring [61]. Taken together, these principles explain why regulatory translation is slow: heterogeneous datasets, non-standardized annotations, limited explainability, and the absence of prospective multicentre evidence make it difficult to demonstrate the reproducibility and safety needed for routine clinical use.

For these reasons, the establishment of a multicentre collaborative database should be considered a priority rather than a future option. Such an infrastructure should prospectively collect raw DICOM data from CT, MRI, PET/CT, and, where feasible, ultrasound; preserve acquisition and reconstruction metadata; include harmonised tumour and lymph-node annotations; and link every case to pathology, sentinel-node findings, surgical reports, follow-up, and clinically meaningful outcomes. Equally important, the database should adopt a common data dictionary, a shared annotation manual, quality-control rules for imaging and labels, and predefined external-validation splits by institution, scanner vendor, and time period. A federated or privacy-preserving architecture could further facilitate participation by centres unable to transfer imaging data directly. This type of collaborative platform would make it possible to test calibration drift, subgroup performance, uncertainty estimates, and true external reproducibility, while also creating the evidentiary base needed for regulatory assessment. In this sense, the next step for AI in OSCC is not merely to produce another accurate retrospective model, but to move from isolated proof-of-concept studies to standardized, prospectively curated, multicentre evidence capable of supporting clinical decision-making.

Beyond purely image-based AI models, multivariable predictive tools may represent an intermediate step toward individualized risk stratification. In this context, Shen et al. [62] proposed a nomogram model for predicting LNM in OSCC based on multiple risk factors, including age, sex, lymph node size, tumour size, T stage, differentiation grade, lymphovascular invasion and perineural invasion. Applied to 158 patients with oral cancer, the model achieved an accuracy of approximately 87% and a NPV of 88%. Although this experience was limited by patient heterogeneity and was not specifically restricted to T1–T2 cN0 tumours, it supports the idea that integrated predictive models may help refine the selection of patients for END, SLNB or surveillance.

Notably, in the Shen model, lymph node size assessed by MRI had an important influence on prediction, whereas other imaging modalities were not included [62]. This observation suggests that future models should not rely on a single imaging source, but should integrate broader imaging information from CT, MRI, PET/CT, ultrasound and, where available, lymphatic mapping techniques. A multimodal model combining clinical risk factors, histopathological variables, conventional imaging, radiomics and AI-based segmentation could provide more specific and clinically useful risk stratification for early-stage OSCC.

## Innovative presurgical techniques

Several interesting developments have been presented in recent years related to optical imaging, a non-invasive modality that facilitates the determination of biological structure and composition of tissues and cells by analyzing changes in their optical properties. This process yields detailed images of organs and tissues [63].

Fluorescence imaging is a method of detection in which a target is identified by a specific fluorescence signal. In essence, this process entails the emission of a beam of light of a specific wavelength from a light source, which enters the tissue and results in a significant increase in the fluorophore’s energy levels, leading to its entry into an excited state. So the effect of scattering and absorption on the emitted photons must be considered, and it is this point that is also subject to reflection and refraction at the surface of the tissue. The application of fluorescence imaging can enhance image resolution and could facilitate the distinction between tumor and normal tissue, thereby improving patient care outcomes. The utilization of NIR fluorescent light has recently been incorporated into intraoperative protocols for the purpose of identifying lymph nodes, tumors, and vital structures [64].

An innovative approach was proposed in 2024 by Yuan et al. [65] of the University of California. This study involved a comprehensive examination of the utilization of preoperative CT and intraoperative fluorescence to assess LNMs. The investigation utilized advanced artificial intelligence algorithms on a sample of 46 patients, providing novel insights into the field. The methodology under consideration herein integrates pre-operative and intra-operative data, thereby merging CT and fluorescence information of the primary tumor to complement each other. This approach facilitates the predictive assessment of malignant lymph nodes, with potential future implications for surgical planning and disease treatment [65].

Nishio et al. [66] from Stanford University assessed the efficacy of a systemically injected, NIR fluorescently-labeled, tumor-targeting contrast agent, panitumumab-IRDye800CW, in facilitating the identification of metastatic nodes in an ex vivo setting. In this report, after in vitro photoacoustic imaging characterization, a photoacoustic imaging was performed on excised neck specimens from patients infused with panitumumab-IRDye800CW before surgery. Intraoperative fluorescence imaging was performed before, during, and after neck dissection using the handheld imaging device and optical imaging platform. This pilot study demonstrated the feasibility of a clinical application of photoacoustic imaging using a tumor-specific antibody to identify occult LN metastases in HNSCC patients.

Despite the myriad approaches documented in the extant literature on oral cancer, which frequently exhibit significant disparities and are based on a limited number of patients, the concordance between the various diagnostic approaches, therapeutic strategies, and subsequent histopathological findings on the status of lymph node invasion remains ambiguous. Lin et al. [67] in 2025 published an article investigating the concordance between imaging findings before surgery (preoperative imaging) and pathology results after surgery (postoperative pathology), with the objective of identifying factors that influence imaging accuracy using a retrospective cohort study from 2014 to 2023, with a total of 1,129 medical records. This study shows that preoperative scans taken before surgery for oral cancers are often relatively consistent with the results of tests done after surgery to check for cancer spread to the lymph nodes.

Table 2 presents emerging pre-surgical techniques used to assess or predict SLN involvement in oral cancer. It compares different methods—such as scintigraphy, fluorescence imaging, magnetic tracers, hybrid approaches, and AI-based analysis—by outlining their principles, clinical value, limitations, performance, and availability. Overall, the table highlights how newer techniques aim to improve the detection of occult metastases and guide surgery more accurately. While some methods, like scintigraphy, are already well established, others (such as hybrid tracers and artificial intelligence) show promising results but are still limited by availability, complexity, or the need for further validation.

**Table 2:** Pre-surgical emerging techniques for sentinel lymph node (SLN) assessment or prediction in oral squamous cell carcinoma (OSCC).

**Technique**	**Principle**	**Clinical value**	**Main limitations**	**Performance**	**Availability**
**SLN scintigraphy (^99m^Tc)**	Radiotracer-based lymphatic mapping	Reference standard for SLN biopsy (SLNB); high negative predictive value (NPV)	Nuclear medicine facilities required	NPV ≈ 93%	Medium
**Indocyanine green (ICG) fluorescence [near-infrared (NIR)]**	Optical visualization of lymphatic flow	Radiation-free; real-time intraoperative guidance	Limited penetration depth	Comparable to standard SLNB	Medium
**Superparamagnetic iron oxide (SPIO) tracers**	Magnetic particle uptake in SLNs	Alternative to radioisotopes	Dedicated probes; limited diffusion	Comparable to standard SLNB	Low
**Hybrid tracers (ICG-^99m^Tc)**	Combined radioactive and fluorescent mapping	Higher SLN detection; complementary guidance	Increased procedural complexity	Superior detection rates	Low
**AI/radiomics**	Machine-learning feature extraction	Improved prediction of occult metastases	Retrospective data; limited validation	Accuracy ~90–93%	Experimental

## Perspectives and future directions

The future management of the clinically negative neck in T1–T2 OSCC should move from a single-technique approach toward an integrated, risk-adapted strategy. The evidence reviewed in this article shows that neither conventional imaging nor emerging pre-surgical techniques currently provides a universal solution capable of safely replacing pathological staging in all patients. END remains oncologically reliable, but it may expose a proportion of patients to unnecessary morbidity. Conversely, SLNB offers a less invasive alternative, but requires dedicated expertise, nuclear medicine infrastructure, pathological ultrastaging, structured follow-up and careful patient selection.

For this reason, the central challenge is not simply to identify the most accurate imaging modality, but to define which combination of clinical, pathological, imaging and molecular information can best stratify the individual risk of occult nodal disease. Standard imaging should continue to play a fundamental role in anatomical staging and surgical planning, but its limitations in detecting micrometastatic disease must be acknowledged. Similarly, lymphatic mapping and sentinel node procedures may refine staging, but their effectiveness depends on standardized workflows and multidisciplinary coordination.

Artificial intelligence, radiomics, fluorescence-guided techniques, hybrid tracers and multimodal imaging integration may progressively refine nodal risk assessment. However, their future value will depend on whether they can improve real clinical decision-making, rather than merely increase diagnostic accuracy in retrospective datasets. To be clinically meaningful, predictive models should demonstrate calibration, external reproducibility, decision-curve benefit and measurable impact on patient management. A model with high diagnostic accuracy but poor calibration or uncertain clinical utility may be scientifically interesting, but it remains insufficient to guide surgical decisions.

Prospective multicentre validation, standardized acquisition protocols, shared annotation criteria and predefined clinical endpoints are therefore essential before these tools can be incorporated into routine OSCC management. In this perspective, multicentre collaborative databases integrating imaging data, clinical variables, pathology, sentinel-node findings, surgical reports and follow-up outcomes should be considered a key prerequisite for developing reproducible and clinically transferable models. Ideally, these collaborative platforms should also include patients from both high-volume referral centres and peripheral or underserved settings, so that future diagnostic pathways are not only accurate, but also scalable and equitable.

The real innovation is therefore not the replacement of conventional imaging by artificial intelligence, but the transformation of imaging from a purely morphological tool into a quantitative, biologically informed and clinically actionable decision-support system.

## Conclusions

In recent years, knowledge of OSCC biology, tumour aggressiveness and lymphatic spread has increased substantially. At the same time, conventional imaging, lymphatic mapping, optical guidance and AI-based methods have expanded the range of tools potentially available for the management of the clinically negative neck. Nevertheless, preoperative nodal staging in T1–T2 OSCC remains a major unresolved challenge.

Conventional imaging techniques, including ultrasound, CT, MRI and FDG-PET/CT, remain essential for anatomical staging, treatment planning and the detection of clinically evident disease. However, their NPV is still insufficient to safely exclude occult nodal metastases in all patients, especially when metastatic involvement occurs in normal-sized lymph nodes or as micrometastatic disease.

SLNB currently represents the most mature individualized approach for selected patients, as it can reduce unnecessary END while preserving accurate pathological staging. However, its implementation requires nuclear medicine availability, surgical expertise, pathological ultrastaging and reliable surveillance. Emerging approaches, including fluorescence imaging, hybrid tracers, magnetic tracers, radiomics and AI-based models, are promising, but still require stronger prospective evidence, external validation and demonstration of clinical utility before they can be adopted in routine practice.

Overall, no single technique currently emerges as a universal solution. The most realistic strategy is an integrated, multidisciplinary pathway combining clinical assessment, tumour pathology, conventional imaging, lymphatic mapping and validated predictive tools. In this framework, the goal is not simply to “save the neck” in all early-stage OSCC patients, but to identify more precisely which patients can be safely spared END without compromising oncological outcomes.

## Abbreviations

CECT: contrast-enhanced computed tomography
CE-MRI: contrast-enhanced magnetic resonance imaging
DWI: diffusion weighted imaging
END: elective neck dissection
FDG-PET: fluorodeoxyglucose positron emission tomography
ICG: indocyanine green
LNMs: lymph node metastases
NACT: neoadjuvant chemotherapy
NIR: near-infrared
NPV: negative predictive value
OSCC: oral squamous cell carcinoma
PPV: positive predictive value
SLN: sentinel lymph node
SLNB: sentinel lymph node biopsy
SPECT/CT: Single Photon Emission Computed Tomography combined with Computed Tomography scans
SPIO: superparamagnetic iron oxide
US: ultrasonography

## Declarations

### Copyright

© The Author(s) 2026.
